# Iron Homeostasis in Health and Disease

**DOI:** 10.3390/ijms17010130

**Published:** 2016-01-20

**Authors:** Raffaella Gozzelino, Paolo Arosio

**Affiliations:** 1 Inflammation and Neurodegeneration Laboratory, Chronic Diseases Research Center (CEDOC), Nova Medical School (NMS)/Faculdade de Ciências Médicas, University of Lisbon, Lisbon 1150-082, Portugal; 2 Department of Molecular and Translational Medicine (DMMT), University of Brescia, Brescia 25123, Italy

**Keywords:** iron, iron metabolism, iron toxicity

## Abstract

Iron is required for the survival of most organisms, including bacteria, plants, and humans. Its homeostasis in mammals must be fine-tuned to avoid iron deficiency with a reduced oxygen transport and diminished activity of Fe-dependent enzymes, and also iron excess that may catalyze the formation of highly reactive hydroxyl radicals, oxidative stress, and programmed cell death. The advance in understanding the main players and mechanisms involved in iron regulation significantly improved since the discovery of genes responsible for hemochromatosis, the IRE/IRPs machinery, and the hepcidin-ferroportin axis. This review provides an update on the molecular mechanisms regulating cellular and systemic Fe homeostasis and their roles in pathophysiologic conditions that involve alterations of iron metabolism, and provides novel therapeutic strategies to prevent the deleterious effect of its deficiency/overload.

## 1. Introduction

**Figure 1:**
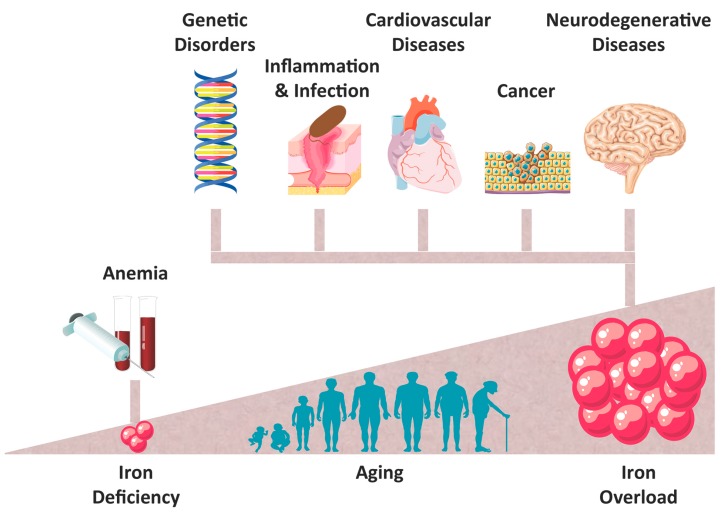
The importance of Iron in pathophysiologic conditions. Essential to ensure survival, disruption of iron homeostasis has been shown to be involved in a variety of pathophysiological conditions, which include anemia and iron-overload related disorders. In particular, the importance of tissue iron accumulation in inflammation and infection, cancer, genetic, cardiovascular and neurodegenerative diseases continuously increases.

Iron (Fe) is a trace metal essential to ensure the survival of almost all organisms. Its participation in heme- and iron-sulfur cluster (ISC)-containing proteins allows the involvement of Fe in a variety of vital functions, including oxygen transport, DNA synthesis, metabolic energy, and cellular respiration [1,2]. However, the ability of Fe to exchange single electrons with a number of substrates can lead to the generation of reactive oxygen species (ROS), as a result of Fe participation in the Fenton chemistry [3]. This triggers oxidative stress, lipid peroxidation, and DNA damage, which can lead to genomic instability and DNA repair defects [4,5] that ultimately compromise cell viability and promote programmed cell death (PCD) [6]. Under physiologic conditions these deleterious effects are prevented by fine-tuned regulatory mechanisms, which maintain systemic and cellular Fe homeostasis [7] through the cooperation of functional compartments (erythroid and proliferating cells), uptake and recycling systems (enterocytes and splenic macrophages), storage elements (hepatocytes), and mobilization processes that allow Fe trafficking through polarized cells and their corresponding organs, presumably assisted by the poly-r(C)-binding protein-1 (PCBP-1)-mediated transport [8,9]. The treatments of pathological conditions associated with Fe overload strongly improved with the introduction of Fe chelators and the advance of chemical, immunohistochemical, scanning transmission X-ray microscopy, and magnetic resonance imaging (MRI), important tools to monitor these conditions. Synchrotron X-ray fluorescence (SXRF) and/or absorption (XAS) are among the best available techniques to determine Fe forms and tissues distribution [10]. In addition, MRI is a powerful tool to detect and assess the size of insoluble Fe deposits (hemosiderin) [11], although its sensitivity is not sufficient to visualize other potentially toxic forms of Fe, such as labile Fe or excess of cytosolic or soluble ferritin [12]. Growing efforts are being made to correlate the results obtained from spatial analyses with SXRF and XAS, and the distribution of Fe observed by MRI. These techniques mainly used in the diagnosis of hepatic disorders, neurodegenerative [10] and cardiovascular diseases [11], will probably be extended to other organs after further investigations. The growing number of data showing a role of Fe in pathologic conditions was a stimulus to this review (Figure 1).

## 2. Iron Metabolism

In healthy individuals, the amount of body Fe is maintained within a range of 4–5 g [13] by a strict control of its absorption, mobilization, storage, and recycling. Fe excretion is not actively controlled and skin desquamation is the major mechanism described so far, accounting for about 1–2 mg per day [14]. Contrarily, well studied are the processes of Fe uptake and recycling that supply the daily need for hemoglobin (Hb) synthesis (25–30 mg). Degradation of senescent red blood cells (RBCs) by splenic macrophages accounts for 90% of total Fe recycling, the remaining 10% comes from the diet [13]. This process occurs in the duodenum where the duodenal cytochrome b ferrireductase (Dcytb) reduces it to Fe(II), which is then offered to the divalent metal transporter-1 (DMT-1) for cellular uptake [15]. DMT-1 (SMF-3 in *C. elegans*) is located on the apical membrane of duodenal epithelial cells and is involved also in Fe reabsorption from glomeruli filtration, recycling from RBCs and transfered from endosomal to cytosolic compartments [16]. The important role of DMT-1 is revealed by the pathologic conditions caused by its mutations, including a severe form of microcytic hypochromic anemia extensively studied in humans and in the Belgrade rat [17].

Inside the cell, the Fe levels are controlled by an elegant machinery involving the Iron Regulatory Proteins (IRPs) and the Iron Responsive Elements (IREs). In low Fe conditions, the high affinity binding of IRPs to IREs inhibits mRNA translation when located in 5’UTR (e.g., Ferritins) and stabilizes the mRNA when in the 3’UTR (e.g., transferrin receptor 1, TfR1). In conditions of Fe excess, ferritins are derepressed and TfR1 is downregulated [18]. This Fe-mediated post-transcriptional regulation modulates the expression of a series of proteins responsible for internalization and storage or utilization of this metal. This ensures that when in excess, Fe is readily stored within the multimeric ferritin made of H- (FtH) and L-chains (FtL), from which is released to satisfy possible intracellular demands [19]. This system, partially conserved in animals, in *C. elegans* involves a cytosolic aconitase (ACO-1) activated in response to hypoxia, while ferritin expression is regulated via activation of the insulin/insulin-like (IIS) growth factor signaling pathway and is favored by increased intracellular stress [16].

Cellular Fe export is mediated by Ferroportin-1 (Fpn1), the only Fe exporter described so far [20]. Its function to release Fe into circulation is supported by the ferroxidase activity of multicopper proteins, namely hephaestin or ceruloplasmin (CP), that favor the formation of Fe-transferrin (Tf) complexes [12]. These bind to cellular surface TfRs for endocytosis and the Tf-Fe^3+^ complex is subsequently reduced by STEAP3 ferrireductase, a step necessary to deliver Fe to mitochondria for heme synthesis and ISC formation [21,22].

It was more recently shown that systemic Fe homeostasis is ensured by a body sensor, hepcidin, a peptide hormone produced by the liver in response to Fe and inflammation. It is controlled also by erythroferrone (ERFE), an erythroid protein produced upon erythropoietic stimulation that suppresses the synthesis of hepcidin [23]. Hepcidin acts as negative regulator of Fe uptake by binding the Fe exporter Fpn1, promoting its internalization and subsequent degradation [24], consequently, increased levels of ERFE enhance Fe availability and release the inhibitory effect of hepcidin on Fe absorption [25]. Indeed, upregulation of hepcidin prevents duodenal Fe uptake and entry into circulation, recycling from RBC heme-Fe and release from Fe stores. The important role of hepcidin in Fe metabolism leads to considering it one, if not the main, regulator of Fe homeostasis [26].

## 3. Heme-Iron Regulation

Most of the body Fe is contained within the protoporphyrin ring of heme, an insertion catalyzed by the mitochondrial enzyme ferrochelatase. Heme acts as prosthetic group in a variety of proteins essential for cellular proliferation, differentiation, and proper functioning, named hemoproteins, which include globins, cytochromes, myeloperoxidases, catalase, and guanylyl cyclase among others [27].

Similarly to Fe, accumulation of non-hemoprotein bound heme (referred as free heme) is cytotoxic, as sensitizes tissue parenchyma cells to undergo apoptosis in response to pro-inflammatory agonists [28,29]. Disruption of heme homeostasis in porphyrias, anemia, hemolytic diseases, hyperuricaemia, intracerebral hemorrhage and neurological disorders, atherogenesis, were demonstrated by its deficiency/accumulation. In addition, the ability of heme to elicit cytokine production, vascular permeability, and recruitment of immune cells to the inflamed tissue confirm its participation in the first stages of inflammatory conditions [30].

The deleterious effect of free heme relies on the capacity of Fe to participate in the Fenton chemistry. Thus, the maintenance of heme homeostasis is essential to prevent its cytotoxicity and, as such, the existence of a systemic and intracellular regulation is fundamental [31]. Two main circulating proteins are upregulated in response to stressful conditions and afford protection against the pro-oxidant effect of heme. Haptoglobin (Hp) immediately captures the cell-free Hb released in circulation from RBCs disruption, thus preventing its further oxidations, while hemopexin (Hx) is a heme scavenger that inhibits the cellular entry of free heme, delivering it safely to macrophages and hepatocytes for Fe recycling [32,33]. A number of membrane heme transporters were discovered so far to play an important role in heme homeostasis. Two Feline Leukemia Virus subgroup C cellular Receptor (FLVCR) isoforms control heme extracellular trafficking, FLVCR1a is essential for skeletal formation and vascular integrity, and FLVCR1b avoids heme accumulation during fetal erythropoiesis [34,35]. The ubiquitous location of ATP-Binding Cassette, subfamily G, member 2 (ABCG2) at the apical membrane of the cells also facilitates heme extracellular export and prevents the deleterious effects of its accumulation [36,37]. Intracellular heme trafficking is mainly mediated by the heme-importer heme-responsive gene 1 (HRG-1) [38], fundamental to ensure the recycling of this molecule by erythrophagocytic macrophages [39]. Roles of heme importers have also been proposed for the heme carrier protein 1 (HCP1), ABCB6, and FLVCR2 [40].

## 4. Genetic Disorders—Hemochromatosis

The importance of Fe in pathophysiologic conditions continuously increases [6] and the first type of inherited disorders in which the contribution of this metal was well-established is hereditary hemochromatosis (HH), caused by mutations in genes maintaining Fe homeostasis. Different types of HH have been discovered so far and the severity of the phenotypes observed varies with the gene(s) involved [41]. The most common type of HH (type I) is adult onset and accounts for >80% of all hemochromatosis patients, mostly Caucasian. It is caused by mutations in the hereditary hemochromatosis (HFE) protein and the most prevalent substitution is cysteine 282 to tyrosine. This leads to the inability of HFE to sense increased levels of Fe and interact with TfR1 [42], which causes decreased hepcidin expression, Fe overload, and possible liver dysfunction. The rare type III HH has a similar phenotype, but caused by mutations in TfR2 gene that abolish its capacity to sense Fe levels and interact with HJV and HFE in the liver. This results in a decrease of hepcidin expression and increase of Fe accumulation in the liver and heart. Type II (juvenile HH) is a more severe disorder that affects younger individuals and causes a fast and heavy Fe overload in the liver and parenchyma. If left untreated, it leads to Fe-mediated multi-organ dysfunction. It is associated with mutations in hemojuvelin (HJV) or hepcidin (type II a and type II b, respectively) and with extremely low levels of serum hepcidin. Type IV HH (ferroportin disease) differs from the other ones for having an autosomal dominant transmission and for not affecting hepcidin expression. It is caused by mutations in the SLC40A gene, which encodes the Fe exporter Fpn, namely the hepcidin target [43]. Known also as ferroportin disease, this is characterized by hyperferritinaemia, normal Tf saturation, and Fe-loaded macrophages [44]. Several mutations have been described, most cause a loss of function, while in few cases they cause a gain of function that results in high serum Ft concentration, elevated Tf saturation, and accumulation of Fe in parenchymal cells [15,41,44,45]. Among the various mutations, a characterized one is the Cys326 to Ser substitution [46], which abolishes a ferroportin crucial binding site to hepcidin [47]. The introduction of C326S in the endogenous ferroportin locus in a knockin mouse confirmed the phenotypic pattern of hepatic damage, increased risk of cirrhosis, and hepatocellular carcinoma [48] observed in human type IV HH, which is caused by an unrestrained Fe export from macrophages and enterocytes, and a lethal exocrine pancreatic function [47,49].

## 5. Iron Deficiency and Anemia

When reviewing the importance of Fe in pathophysiologic conditions it is common to refer mainly to Fe overload and its deleterious effects. However, we should remind that disruption of Fe homeostasis also implies a decrease in its level and subsequent biological consequences. Fe deficiency is the most common disease worldwide and affects mainly children, women, and the elderly [50]. Decreased Fe levels may be associated with mild or severe anemia, conditions that develops when body Fe stores are depleted and the supply of Fe to organs becomes compromised [51,52]. Various causes may contribute to this condition, including genetic defects in proteins responsible for DNA repair, as in Fanconi anemia [4,5,53], and rare genetic mutations occurring in *TMPRSS6* (alias matriptase-2) that cause iron refractory iron deficiency anemia (IRIDA). *TMPRSS6* is a negative regulator of hepcidin expression [45] and its single nucleotide polymorphisms positively correlate with low serum Fe and serum ferritin [54]. Anemic conditions are often caused when increased Fe demand do not meet the adequate supply and occur mainly in children and pregnant women. In addition, enhanced Fe loss affects mostly elderly and results from the use of drugs that cause gastrointestinal bleeding [55]. A contributor to Fe deficiency in elderly comes also from the continuous exposure to subclinical inflammation, which, as described below, modulates hepcidin expression, impairs the maintenance of Fe homeostasis, and leads to the development of anemia of chronic diseases (ACD) [56]. Novel therapeutic approaches to correct the anemia due to hepcidin excess are still under investigation. Among them, the ablation of ERFE in mice was shown not to improve inherited anemias with high hepcidin and ineffective erythropoiesis [57], while heparin administration effectively reduced hepcidin expression. In mice, the therapeutic use of heparins without anticoagulant activity increased the levels of serum Fe, ameliorating significantly the clinical symptoms associated with anemia of inflammation and IRIDA. These results strongly suggest that endogenous heparan sulfate proteoglycans contribute to regulate hepcidin expression. Humanized hepcidin monoclonal antibodies as well as structured l-oligoribonucleotide are currently tested in clinical trials for the treatment of cytokines-induced hypoferremia, aimed to prevent ferroportin–hepcidin interaction and to reduce hepcidin expression [58].

## 6. Iron and Cancer

Fe homeostasis is altered in most cancer patients, who are affected by anemia in more than 40% of cases [59] and up to 90% when undergoing chemotherapy [60]. The low availability of systemic Fe is partially promoted by the tumor itself that sequesters the metal to ensure its proper growth [61]. In fact, Fe availability is fundamental for cell proliferation and thus highly up-taken by neoplastic cells [62]. Changes in Fe metabolism characterize all phases of tumor development, from proliferation to metastasis [4,62]. Abnormal expression of TfR1 and FtH [63,64,65] are observed in a variety of cancers, as well as increased levels of circulating hepcidin [64], and these proteins are potential clinical predictors for the prognosis of lung, breast, prostate, liver, and pancreatic cancer [66,67,68,69]. The importance of Fe in tumor development has been confirmed in solid and blood tumors, including lymphoma, and multiple myeloma [70,71], where adverse prognosis correlates to Fe load in the affected tissue. The Fe deregulation in tumor cells is associated with ROS production, which promotes DNA modification, strand breaks and is potentially mutagenic [72,73,74,75]. Among the effects of the oxidative stress generated by Fe accumulation, which are relevant to malignant transformation, there is the activation of signaling transduction pathways that are essential for tumor growth [67]. These include p53, Wnt, NF-κB, Hypoxia-inducible factor (HIF), DNA replication and repair, cyclins and cell cycle regulation, AKT, and epidermal (EGF) and vascular endothelial growth factor (VEGF) [61,67]. Recent studies also indicate the capacity of Fe-driven ROS to induce epigenetic changes that favor tumor metastasis, as they trigger mutations in hot spots and the suppression and/or activation of tumor suppression genes and/or proto-oncogenes, respectively [4,5,61,67,75]. The inflammation associated with cancer also contributes to Fe deregulation and promotion of DNA damage. Tumor immunology is one of the most investigated fields known to play a dominant role in tumor growth [76] and also aimed to the development of therapeutic strategies eliciting anti-tumor responses [77,78]. Further investigations may be required to assess whether the potential combination of Fe chelators and immune therapy could prevent tumor growth and/or relapse.

## 7. The Iron-Inflammation Connection

Inflammation plays a critical role in controlling Fe metabolism, as the pro-inflammatory cytokines released upon immune cell activation alter the levels of proteins regulating Fe homeostasis [79]. Taking into account that Fe is essential for proliferation of both prokaryotes and eukaryotes, disruption of its homeostasis may either favor the establishment of the infection or act as a host defense mechanism to defeat pathogen invasion [80,81]. The ability of Fe to impair cytokine secretion renders individuals with Fe overload more susceptible to systemic infections than those with Fe deficiency, therefore excess Fe favors pathogen competition for Fe and increases the morbidity and mortality of infectious diseases [82]. An interesting hypothesis that was recently proposed is that macrophages and T-lymphocytes take up and accumulate non-transferrin bound Fe (NTBI), thus acting as circulating Fe storage compartments to protect different organs from Fe-dependent cytotoxicity [12,83]. The engagement of pattern recognition receptors (PPRs) and the release of pro-inflammatory cytokines (IL-6, IL-22, Oncostatin-M or Activin B) from these immune cells stimulate hepcidin expression, which is considered one of major host defense strategies against infection, an effect triggered by restricting Fe availability to pathogens [84,85]. Macrophage Fe retention is protective in most of the cases, but it may also inhibit protective anti-microbial strategies, which become no longer effective to prevent pathogen growth. This is the case for intracellular invading organisms, as *Salmonella typhimurium*, able to induce hepcidin expression and cellular Fe to ensure its own growth [84]. Much studied is the role of hepcidin in malaria, in which high levels are associated with diminished hepatic growth and differentiation of *Plasmodium* parasite [86]. Fe deficiency was shown to correlate with decreased malaria susceptibility in mice and humans, and consistently excess Fe positively correlates with increased lethality. This notion is also supported by the observation that, in a randomized scale trail, most children succumbing to the infection were the ones receiving Fe supplementation [51,85]. Mouse studies indicate that the disruption of Fe homeostasis could also underlie the poor outcome observed in pregnancy malaria, which results in abortions, stillbirths, underweight babies, and fetal and maternal mortality. The characteristic microvasculature of the placenta, composed of high and low blood flow regions, seems to favor the sequestration of infected RBCs, the lysis of which is a cause of heme/Fe-mediated cytotoxicity to trophoblasts and the fetus. While this would contribute to explain the correlation between Fe overload and the severity of placental malaria (PM), further investigations are required to assess whether this is due to a dysfunctional intracellular and extracellular heme/Fe trafficking [87]. An evolutionary defense strategy against malaria infection is provided by the inheritance of one copy of defective beta globin gene, which confers survival advantage against this disease. However, individuals carrying the two copies of the mutations and suffering from sickle cell disease (SCD) develop vaso-occlusion, endothelial cell dysfunction and chronic vasculopathy, all symptoms mediated by heme/Fe driven cytotoxicity that cannot be prevented by the low level of Hp and Hx plasma proteins observed in these conditions. Therapeutic approaches based on the re-establishment of Fe homeostasis, achieved by overexpression of FtH, are currently under investigations in mice, in which increased levels FtH were shown to prevent Hb-mediated microvascular stasis and ameliorate the symptoms associated [88].

## 8. Role of Iron in Cardiotoxicity

The involvement of inflammation in cardiotoxicity was extensively demonstrated in cancer patients, in which severe cardiomyopathy remains a major concern of chemotherapeutic drugs administration. The involvement of Fe metabolism in the adverse effects induced by anthracycline and doxorubicin (DOX) treatment, as cardiomyopathies and congestive heart failure, was confirmed by the protective role of Fe chelators. Preventing disruption of Fe homeostasis and restoring the normal expression of Fe transporters, Fe chelator therapy in anthracycline-induced cardiotoxicity suppresses the deleterious effects of Fe overload, of which its involvement was also demonstrated by the increased susceptibility to this treatment of HFE-deficient mice [89]. Whether the use of Fe chelators may also be applied to prevent common cardiac failure remains to be established [11]. Nevertheless, the role of Fe in cardiovascular diseases (CVD) was extensively demonstrated in epidemiological studies, reporting the existence of a positive correlation between Fe accumulation and CVD [90]. This notion was further supported by the lower incidence of ischemic heart disease in Fe deficient patients. The association between disruption of Fe homeostasis and atherosclerosis-driven ischemic CVD was first postulated by Sullivan in 1980s, who indicated that increased Fe levels may enhance the risk of CVD. This may explain the enhanced susceptibility to CVD of post-menopausal women when compared to pre-menopausal [91,92], for whom a reduced level of hepcidin was assumed to diminish more than 50% the risks of CVD when compared to men of the same age. In agreement with this observation, a number of investigations confirmed the protective effect of blood donations against CVD, as this reduces body Fe stores and improves vascular function [93]. The importance of Fe in cardiac function has been demonstrated also in studies using mice that were genetically deleted for TfR-1 or FPN in cardiomyocytes and shown to develop severe cardiomyopathy and heart failure [94,95]. While this is due to a phenotype associated with a heart Fe deficiency or Fe overload, respectively, the restoration of Fe homeostasis via supplementation or chelation of this metal further confirms its dominant role in the development of CVD. Although little is known about cardiac Fe utilization, the ratio between soluble TfR and serum ferritin has been shown indicative of acute myocardial infarction. In particular, the levels of serum ferritin were demonstrated to directly correlate with the incidence and progression of atherosclerosis, which leads ultimately to the occurrence of CVD [93,96,97,98,99,100]. This notion is further confirmed by the increased expression of FtH and FtL in atherosclerotic lesions. The involvement of Fe in modulating the activity of enzymes fundamental in the regulation of cholesterol and triglycerides as well as in the induction of low-density lipoprotein (LDL) oxidative modifications leads to formation of Fe-rich macrophages, *i.e*., foam cells, responsible for atherosclerotic plaque development, progression and subsequent vulnerability for rupture [90,93,101]. Degradation of the cell-free hemoglobin subsequently to intraplaque hemorrhages results in the release into circulation of its heme prosthetic groups promotes unfettered production of free radicals and oxidative stress, strongly involved in the development of atherosclerotic lesions. The cytotoxicity induced by continuous release of heme in circulation, as in thalassemic and SCD suffering individuals, is apparently the leading cause of CVD, a belief also confirmed by the protective effect of the Hb/heme scavenger Hp and the heme degrading enzyme heme oxygenase 1 (HO-1) in the development of these pathologies. Interestingly, a strong correlation was found between Hp polymorphic genotype and the level of Fe within atherosclerotic plaques, macrophage infiltration, and plaque instability, all symptoms associated with enhanced CVD risks. Moreover, the anti-oxidant and vasodilator properties of HO-1 prevented heme/Fe-mediated endothelial dysfunction and atherogenic plaque formation [93,101]. The higher occurrence of cardiovascular death and non-ischemic cardiovascular abnormalities in hemochromatosis patients as well as the role of hepcidin in Fe retention by macrophages and their consequent transformation into foam cells is currently under investigation. Contrarily to the expectations, hepatic hepcidin expression does not seem to correlate with atherosclerosis progression, as increased accumulation of Fe in macrophages of atherosclerotic mice does not promote atherosclerotic lesions or calcification. However, despite the debate about whether Fe loaded macrophages would aggravate atherosclerosis-mediated CVD, the beneficial effect of the Fe chelator deferoxamine on the recovery of ischemia/reperfusion-induced animals and the outcome of patients submitted to coronary artery bypass surgery [90] reveals the crucial role of Fe in the pathogenesis of CVD.

## 9. Iron, Aging, and Neurodegeneration

The pathologies mentioned above imply systemic and local dysregulations of Fe homeostasis, but it is still not clear whether this is also the case for neurodegenerative diseases, in which Fe overload is postulated as one of the main contributors to neuronal death. Fe is required for normal brain functions, e.g., neural respiration and metabolic activities, myelin synthesis, production of neurotransmitters and synaptic plasticity [102]. The importance of brain Fe homeostasis is also indicated by the retardation and impaired cognitive abilities caused by its deficiency during early development as well as the axonal degeneration and neuronal death triggered by its overload [13]. Fe physiologically accumulates in aging brains via a mechanism that remains to be fully elucidated and that is accompanied by a reduced expression of anti-oxidant proteins and repair mechanisms, which contribute to Fe-mediated oxidative stress [102,103]. During aging, the disruption of Fe homeostasis affects all organs and contributes to the senescence process. Nutritional habits could contribute to increase Fe content, and meta-analysis studies indicated a positive correlation between elevated Fe consumption and increased incidence of age-related pathologies [71]. This notion is also supported by mouse experiments demonstrating that animals fed with an enriched Fe-diet senesce faster than those maintained under a restricted Fe diet. The high level of inflammation and circulating hepcidin, observed under these conditions, is accompanied by disrupted Fe homeostasis, altered levels of Fe, and organs senescence, as particularly evident in liver, spleen, and gut [103,104]. However, it remains poorly understood how increased dietary Fe uptake affects the brain. Nevertheless, it is well known that the levels of Fe increase disproportionally in neurodegenerative diseases, e.g., Parkinson’s, Alzheimer’s, Huntington’s, Prions, and neurodegeneration with brain Fe accumulation (NBIA), when compared to brains of elderly individuals [6,103,105]. It is established the involvement of this metal in Friedrich’s ataxia, a disease characterized by progressive degeneration of sensory neurons in the dorsal root ganglia caused by a mutation in the frataxin gene. Frataxin plays an essential role in delivering Fe to mitochondrial pathways involved in ISC biogenesis [5] and when its functionality is reduced below a critical threshold, Fe accumulates in mitochondria [106]. Reduced mitochondrial functioning and impaired electron transport chain results in Fe-mediated ROS, which was shown to positively correlate with the severity of Friedrich’s ataxia. Mitochondrial defects are also associated with the pathogenesis of Parkinson’s disease (PD) [107], in which a compromised Fe transport to this organelle leads to its accumulation and contributes for the formation of alpha-synuclein aggregates, a hallmark of PD. Disruption of Fe homeostasis is also observed in patients suffering from early onset Alzheimer’s disease (AD), where brain and cerebrospinal fluid (CSF) accumulation [108] promotes β-amyloid aggregation and hyperphosphorylated tau, two typical features of AD [105]. While different expression or mutations in genes regulating Fe metabolism underlie a number of neurodegenerative diseases, including restless syndrome, neuroferritinopathies, and aceruloplasminemia [109,110], the requirement of Fe also in maintaining lipids homeostasis may suggest a potential role for this metal in demyelinating conditions. However, the observation that most of the clinical symptoms associated with these diseases occur in adulthood implies the existence of protecting mechanisms that retard/prevent brain Fe toxicity. Whether the deleterious effect of brain Fe accumulation is associated with exposure to sub-lethal chronic or acute inflammation is currently the object of investigations [102]. A high level of pro-inflammatory cytokines has been detected in the affected brain regions and they may regulate local Fe homeostasis [111]. In addition, systemic inflammation significantly increases the expression of brain hepcidin, suggesting the existence of a hepcidin/ferroportin axis that actively regulate Fe in the brain. Whether the FPN1/hepcidin axis may be involved in a potential redistribution of Fe between the peripheral and central compartment remains to be elucidated [58,102]. Detoxification of brain Fe overload is a task pursued by Fe chelators, most of which have poor access to the brain. Promising results were obtained in clinical trials with deferiprone, a compound able to translocate across the blood brain barrier and scavenge excess Fe from regional foci of siderosis. By efficiently relocating this metal, deferiprone replenishes Fe-deprived regions and prevents the deleterious effects of Fe maldistribution [12,112].

## 10. Conclusions

Major advances in the understanding of the mechanisms regulating intracellular and systemic levels of Fe proved that the alteration of Fe homeostasis in mammals underlies a variety of pathological conditions. The oxidative damage generated by the participation of Fe in redox reactions was shown to be the leading cause of programmed cell death and tissue damage, which might also be enhanced by the improper compartmentalization of this metal rather than its total accumulation. Now, the challenge is in developing new approaches capable to restore the deregulation of Fe that accompanies and exacerbates these disorders, which are capable of specifically targeting the tissues involved.

## Abbreviations

Fe: Iron
ISC: Iron-Sulfur Cluster
ROS: Radical Oxygen Species
PCD: Programmed Cell Death
PCBP: Poly-r(C)-Binding Protein 1
MRI: Magnetic Resonance Imaging
SXRF: Synchrotron X-ray Fluorescence
XAS: X-ray Absorption Spectroscopy
RBCs: Red Blood Cells
Dcytb: duodenal cytochrome b ferrireductase
DMT-1: Divalent Metal Transported 1
IRP: Iron Regulatory Protein
IRE: Iron Responsive Element
TfR-1: Transferrin Receptor 1
FtH: Ferritin H Chain
FtL: Ferritin L Chain
ACO-1: Aconitase 1
Fpn: Ferroportin
CP: Ceruloplasmin
Tf: Transferrin
ERFE: Erythroferron
Hp: Haptoglobin
Hx: Hemopexin
FLVCR-1: Feline leukemia virus subgroup C cellular receptor 1
ABCG-2: ATP-Binding Cassette, subfamily G, member 2
HRG-1: Heme Responsive Gene 1
HH: Hereditary Hemochromatosis
HJV: Hemojuvelin
IRIDA: Iron refractory iron deficiency anemia
ACD: Anemia of Chronic Diseases
NTBI: Non-Transferrin Bound Iron
PPRs: Pattern Recognition Receptors
SCD: Sickle Cell Disease
DOX: Doxorubicin
CVD: Cardiovascular Diseases
LDL: Low-Density Lipoprotein
HO-1: Heme Oxygenase 1
NBIA: Neurodegeneration with Brain Iron Accumulation
PD: Parkinson’s disease
AD: Alzheimer’s disease
CSF: Cerebrospinal Fluid
